# Comparison of Hidden Blood Loss in Biportal Endoscopic Spine Surgery and Open Surgery in the Lumbar Spine: A Retrospective Multicenter Study

**DOI:** 10.3390/jcm14113878

**Published:** 2025-05-30

**Authors:** Dae-Geun Kim, Eugene J. Park, Woo-Kie Min, Sang-Bum Kim, Gaeun Lee, Sung Choi

**Affiliations:** 1 Department of Orthopedic Surgery, Soonchunhyang University Gumi Hospital, Soonchunhyang University College of Medicine, Gumi 39568, Republic of Korea; kuroo25@schmc.ac.kr; 2 Department of Orthopedic Surgery, Dream General Hospital, Daegu 702-752, Republic of Korea; 3 Department of Orthopedic Surgery, Kyungpook National University Hospital, Kyungpook National University College of Medicine, Daegu 41944, Republic of Korea; pjj841229@gmail.com (E.J.P.); oswkmin@gmail.com (W.-K.M.); 4 Spine Center, Bogang Hospital, Daegu 42801, Republic of Korea; 5 Department of Orthopedic Surgery, Sejong Chungnam National University Hospital, Chungnam National University College of Medicine, Daejeon 30099, Republic of Korea; sangbumos@icloud.com; 6 Division of Biostatistics, Soonchunhyang University Gumi Hospital, Gumi 39568, Republic of Korea; 20240236@schmc.ac.kr; 7 Department of Orthopedic Surgery, Daegu Fatima Hospital, Daegu 41199, Republic of Korea

**Keywords:** spine surgery, open surgery, biportal endoscopic spine surgery, hidden blood loss

## Abstract

**Background/Objectives**: Biportal endoscopic spine surgery (BESS) is one of the minimally invasive spine surgery techniques. BESS has several advantages, such as better visualization, less muscle injury, early rehabilitation, etc. Due to its clear visualization, delicate intraoperative hemostasis of the bleeding foci, including cancellous bone and small epidural vessels, can be achieved. Therefore, some authors have reported that BESS resulted in less intraoperative visible blood loss (VBL) compared to conventional open surgery. However, it is difficult to analyze the exact amount of intraoperative blood loss because of the continuous normal saline irrigation. In addition, hidden blood loss (HBL) tends to be overlooked, and the amount of HBL might be larger than expected. We aim to calculate the amount of HBL during BESS and to compare our findings with convention open surgery. **Methods**: We retrospectively obtained the clinical data of patients that underwent lumbar central decompression from July 2021 to June 2024. Patients were divided into two groups: the BESS group that underwent biportal endoscopic lumbar decompression, and the open surgery group that underwent open decompression. Both groups used unilateral laminotomy and bilateral decompression techniques. Total blood loss (TBL) using preoperative and postoperative change in hematocrit (Hct) was measured using Gross’s formula and the Nadler equation. Since TBL consists of VBL and HBL, HBL was calculated by subtracting the VBL measured intraoperatively from TBL. **Results**: A total of sixty-six patients in the BESS group and seventeen patients in the open surgery group were included in the study. The TBL was 247.16 ± 346.88 mL in the BESS group and 298.71 ± 256.65 mL in the open surgery group, without significant difference (p = 0.569). The calculated HBL values were 149.44 ± 344.08 mL in the BESS group and 171.42 ± 243.93 mL in the open surgery group. The HBL in the BESS group was lower than the HBL in the open surgery group, without significant difference (*p* = 0.764). **Conclusions**: The TBL and HBL during lumbar central decompression were smaller in patients who underwent BESS compared to those who underwent open surgery. While TBL was significantly lower in BESS, HBL did not show statistical significance between the two groups. HBL during BESS should not be neglected, and related hemodynamics should be considered postoperatively.

## 1. Introduction

Spinal surgery techniques have developed from traditional open spine surgery to minimally invasive spine surgery (MISS). In particular, the field of endoscopic surgery has been rapidly growing in recent decades. While the indications of endoscopic surgery were initially limited to single-level lumbar disc herniations, its application has expanded to multiple-level disc herniations, spinal canal stenosis, spondylolisthesis, instability, and even cervical pathology. Biportal endoscopic spinal surgery (BESS) is one of the endoscopic surgical techniques and was first introduced in 2015. It has gained popularity ever since and has become one of the mainstream means of MISS [1]. BESS has several advantages compared with microscopic open surgery. According to previous studies, BESS resulted in less intraoperative bleeding due to excellent vision of the surgical site and resulted in less hospital stay [2]. However, it is difficult to measure the exact amount of intraoperative bleeding due to the continuous flow of irrigation fluid and blood infiltration into the soft tissue and the dead space of the surgical site [3,4]. Such inaccuracy may result in an unrecognized change in hemodynamics.

The concept of hidden blood loss (HBL) is considered a common problem during joint arthroplasty surgery [5,6]. In orthopedic surgery, HBL was first introduced by Sehat et al. in 2000, and they found that the HBL during total knee arthroplasty and total hip arthroplasty came to 26% and 49% of the total blood loss (TBL) [5]. Recently, several authors have reported their HBL results during MISS. The HBL ranged from 194.4 to 782.4 mL during minimally invasive transforaminal lumbar interbody fusion [7], and the mean HBL in the BESS for herniation of nucleus pulposus (HNP) or lumbar spinal stenosis (LSS) was 361 ± 217 mL, accounting for 77.9% of the TBL [8]. Although the amount of HBL can be overlooked by surgeons, a large amount of HBL could lead to complications related to bleeding [9,10].

To the best of our knowledge, while there are studies on perioperative HBL during BESS, there is no study comparing this with the HBL of open lumbar spine surgery. In this study, we aim to calculate the HBL during BESS and compare it with that of open surgery.

## 2. Materials and Methods

### 2.1. Study Design

This was a multicenter and retrospective study. This study was performed following approval of the Institutional Review Board (IRB number: 2024-13). From July 2021 to June 2024, patients that underwent surgical treatment due to LDH or LSS were retrospectively analyzed. Surgery was performed either by open or BESS according to the surgeons’ preference. We included (1) patients with lumbar radiculopathy or neurogenic claudication; and (2) those not responding to conservative treatment, including medication, nerve block, or physical therapy for at least 3 months before surgery. The exclusion criteria were as follows: (1) age < 18 years old; (2) patients with spine tumor, infection, or trauma; (3) patients with recurrent LDH or a previous spine operation; (4) patients with liver or kidney dysfunction; (5) patients with pathologies other than that of lumbar region. Patients who underwent open surgery were classified as the open surgery group, whereas patients who underwent BESS were classified as the BESS group.

### 2.2. Surgical Technique

#### 2.2.1. Biportal Endoscopic Spine Surgery (BESS) Group

**Figure 1:**
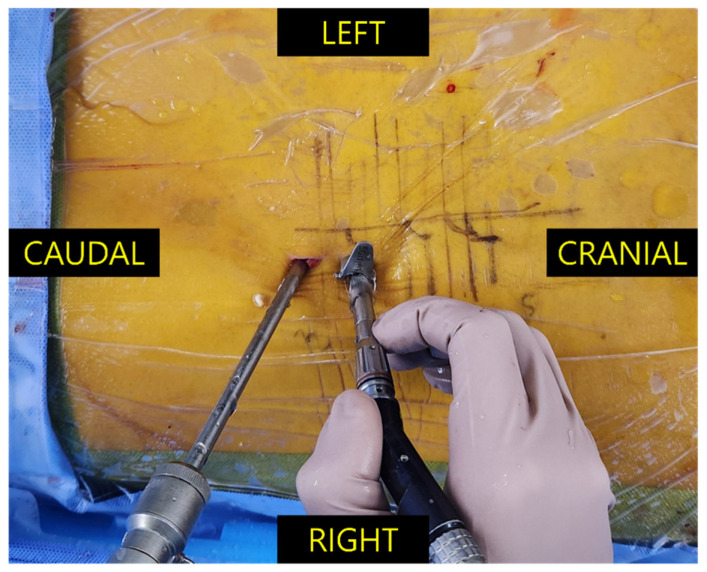
Intraoperative image during biportal endoscopic spine surgery through the right interlaminar space. The left (distal) incision was used as the viewing portal through which the endoscope enters, whereas the right (proximal) incision was used as the working portal through which the surgical instruments are passed. Note the semicircular-shaped tubular retractor placed in the working portal when the high-speed drill is inserted.

Patients were positioned prone after general anesthesia. Two transverse incisions, cutting both the skin and fascia, were made approximately 2 cm apart. After dilating the incisions and soft tissue detachment percutaneously, the endoscope was inserted on one portal, and the working instruments were inserted on the other portal (Figure 1). Unlike open surgery, it was unnecessary to partially removal the spinous process to obtain a wide surgical view. As open surgery, unilateral laminotomy and bilateral decompression were performed. A subfascial drain was inserted before skin closure.

#### 2.2.2. Open Surgery Group

Patients were positioned prone after general anesthesia. A midline skin incision was made, and soft tissues were dissected from bony structures to expose the bony landmarks. A hemilaminectomy retractor was used for retraction, and a surgical microscope was used to visualize the surgical field. If the surgical view was obstructed, the superficial portion of the spinous process was partially removed with a burr. An ipsilateral unilateral laminotomy and flavectomy was performed while avoiding iatrogenic pars fracture. By tilting the microscope and the operating table, contralateral flavectomy/decompression was performed. A subfascial drain was inserted, and the wound was closed layer by layer.

### 2.3. Data Collection

Demographic data, including age, gender, height, weight, and smoking status, were collected through electronic medical records. The length of hospital stay and the intraoperative bleeding loss were also obtained. The intraoperative bleeding amount was measured based on the bleeding amount in the suction bottle subtracted from the amount of irrigation fluid and measuring the blood-tinged gauzes used. The volume in the postoperative drainage was daily recorded until removal. All patients underwent routine blood tests, including hematocrit preoperatively and on the first and/or second postoperative day.

### 2.4. Calculation of Blood Loss

The predicted blood volume (PBV) was calculated via the Nadler equation [11].

- Men: PBV (liter) = (0.3669 × H^3^) + (0.03219 × W) + 0.6041;
- Women: PBV = (0.3561 × H^3^) + (0.03308 × W) + 0.1833;
- H: height (meter); W: weight (kilogram).

Gross has introduced formulae that could calculate the TBL using the PBV and the perioperative change of Hct [12]. We used the preoperative Hct (Hctpre) level closest to the date of surgery and the lowest postoperative Hct level (Hctpost) measured during the hospital stay. By using Gross’s formula, the PBV can be calculated, and the HBL could be calculated accordingly. The formulae are as below:

- TBL (liter) = PBV × (Hctpre − Hctpost)/Hctave;
- Hctpre: preoperative hematocrit level;
- Hctpost: postoperative lowest hematocrit level;
- Hctave: average of the Hctpre and Hctpost.

TBL consists of visible blood loss (VBL) and HBL. VBL is the sum of intraoperative bleeding amount and total postoperative drainage. As a result, HBL can be calculated as follows:

- HBL = TBL − VBL;
- VBL = intraoperative bleeding + postoperative drainage.

### 2.5. Statistical Analysis

The data were statically analyzed using SPSS version 26.0 (IBM Corp, Armonk, NY, USA). Continuous data that followed a normal distribution were presented as mean ± standard deviation, and categorical data were presented as numbers and percentages. An independent samples t-test, Welch’s *t*-test, or Mann–Whitney U test was used to compare continuous data between groups. For categorical data, the chi-square test or Fisher’s exact test was used. A *p*-value below 0.05 was considered statistically significant.

## 3. Results

A total of sixty-six patients in the BESS group and seventeen patients in the open surgery group were included in the study. In the BESS group, the average age of the patients was 66.64 ± 14.18 years, with females comprising 42.4%. In the open surgery group, the average age of the patients was 59.18 ± 10.98 years, and 29.4% of the patients were female. There was no significant difference between the two groups in terms of demographic characteristics (Table 1). No patient required intraoperative packed red blood cell transfusion in either the BESS or the open surgery group.

The mean calculated PBV values for males were 4.34 ± 0.69 L in the BESS group and 4.46 ± 0.46 L in the open surgery group. The mean calculated PBV values for females were 3.93 ± 0.70 L in the BESS group and 4.05 ± 0.46 L in the open surgery group. By using Gross’s formula, the TBL values were 247.16 ± 346.88 mL in the BESS group and 298.71 ± 256.65 mL in the open surgery group. TBL did not show a statistically significant difference between the two groups. The calculated HBL values were 149.44 ± 344.08 mL in the BESS group and 171.42 ± 243.93 mL in the open surgery group. HBL in the BESS group was lower than in the open surgery group but did not show statistical significance (*p* = 0.764) (Table 2).

## 4. Discussion

In previous reports, BESS resulted in less intraoperative bleeding and shorter hospital stay than microscopic decompression [2]. Although BESS is one of the MISS techniques, compared to full endoscopic spine surgery (FESS), it showed inferior results in that it showed more TBL and intraoperative bleeding, longer operation times, and longer hospital stays [13,14].

However, the accuracy of measuring intraoperative blood loss remains controversial. A previous study regarding intraoperative blood loss volume reported that there was a significant difference between the calculated blood loss and the estimated intraoperative blood loss [15]. Also, it is difficult for an anesthesiologist to estimate the exact intraoperative bleeding due to continuous normal saline irrigation during BESS [3]. Although studies reported that MISS techniques show less blood loss than conventional open surgery, HBL was not negligible during MISS. Previous studies reported that the HBL during MI-TLIF was 52.5% of TBL [7]; during OLIF, it was 92.4% of TBL [16]; and during XLIF, it was 79.1% of TBL [17]. A recent study analyzed HBL after unilateral biportal endoscopic (UBE) surgery, which is identical to BESS, and reported diabetes and tissue thickness as independent risk factors of HBL. In their study, HBL after single- and double-level decompression was 257.8 ± 190.66 and 296.58 ± 269.75mL, respectively, which reflects our study results.

HBL contributes to hemoglobin degradation products in tissues, which activate toll-like receptors and promote NF-κB-mediated cytokine release (IL-6, TNF-α) [4,10]. This inflammatory cascade exacerbates postoperative pain and delays functional recovery by sensitizing nociceptors, particularly in diabetic patients where hyperglycemia amplifies responses through advanced glycation end-products. Occult paraspinal muscle hematomas from HBL create mechanical compression and hypoxia, impairing mitochondrial function in type-II muscle fibers [10,18]. This aligns with observations of delayed ambulation in elderly patients despite minimally invasive approaches, as demonstrated in MRI studies showing persistent muscle edema correlating with HBL volume (r = 0.42; p = 0.03) [10,19]. The iron-rich microenvironment from erythrocyte extravasation promotes bacterial biofilm formation, particularly concerning instrumented fusions. Staphylococcus aureus growth increases 3.2-fold in vitro under hematoma-mimetic conditions [18,19]. This risk is compounded in patients with vascular disease or diabetes, where impaired microcirculation prolongs hematoma resorption [19,20].

BESS and FESS are the two mainstream endoscopic spine surgery techniques. According to previous studies, FESS demonstrates less HBL than BESS [14,15]. Compared to BESS, FESS preserves the paraspinal muscle by directly reaching the target point using sequential dilators and a blunt obturator through only one incision [21]. While the muscle detaching and splitting step was identical during BESS and FESS, BESS requires an additional incision for the separate insertion of the endoscope and surgical instruments, which may result in more bleeding [22]. BESS shows a tendency to increase muscle damage compared to FESS due to the process of creating a larger working space in the muscle-lamina interface [23].

In our study results, the TBL and the HBL quantities during BESS were smaller than those during open surgery; however, there was statistically no significant difference. Since the amount of bleeding in BESS is comparable to that in open surgery, this indicates that BESS is more an endoscopic-assisted surgery than a fully endoscopic surgery. Irrigation fluid continuously flows in and out of the surgical field, and such fluid can infiltrate the muscle and subcutaneous layers during BESS. During surgery, the pressure of the irrigation fluid may wash out and mask the bleeding foci rather than actually coagulating the bleeding foci. However, since, unlike open surgery, it lacks a large incision, although not statistically meaningful, the TBL and the HBL quantities were smaller than in open surgery. Although there are several factors underlying postoperative hematoma, we should be wary of postoperative hematoma in cases of BESS.

There are some limitations to the study. At first, the sample size was small. Additionally, the imbalance in sample sizes between the open surgery group (17 patients) and the BESS group (66 patients) necessitates caution in interpreting the statistical analyses. This imbalance may limit the statistical power of the results; therefore, our findings should be considered as preliminary. In the future, a multicenter study with larger samples should be conducted. In our data, the TBL values for some patients were negative. We believe this might be due to the irregular blood testing time points. Blood tests are usually performed when the surgeon and patient decide to undergo surgery at the outpatient visit. Therefore, the predicted blood volume at the time of surgery may have changed compared to the time when the blood test was conducted. Second, the nil per os (NPO) time may vary slightly depending on the order of surgery. Thus, the degree of dehydration may differ between individuals before surgery, which may have affected Hct accordingly. In a future study, a regular perioperative blood testing time should be planned. Also, our study did not stratify HBL impacts by comorbidities like diabetes or vascular disease, which may significantly modulate postoperative outcomes. Future studies should address this demographic variability.

## 5. Conclusions

In the BESS group, the TBL and HBL values were smaller than in the open surgery group. However, the difference in amount was not large, and even in BESS, it was confirmed that there was more TBL and HBL than in full endoscopic surgery. Therefore, the HBL during BESS should not be neglected and should be considered postoperatively. Furthermore, in elderly or comorbid patients, postoperative monitoring should include serial inflammatory markers (e.g., CRP) and neuromuscular assessments to mitigate HBL-related complications.

## Figures and Tables

**Table 1:** Baseline characteristics of both groups.

Characteristics	Total (*n* = 88)	BESS Group (*n* = 66)	Open Surgery Group (*n* = 17)	*p*-Value
Age (years)	65.11 ± 13.86	66.64 ± 14.18	59.18 ± 10.98	0.007
Gender				0.484
Male	50 (60.2)	38 (57.6)	12 (70.6)	
Female	33 (39.8)	28 (42.4)	5 (29.4)	
Height	1.63 ± 0.09	1.63 ± 0.10	1.66 ± 0.06	0.054
Weight	66.71 ± 13.77	66.58 ± 14.65	67.21 ± 10.00	0.534
BMI	24.84 ± 3.79	24.99 ± 3.99	24.23 ± 2.91	0.461
Smoking				0.228
No	69 (83.1)	61 (92.4)	9 (52.9)	
Yes	7 (8.4)	5 (7.6)	2 (11.8)	
Anticoagulant agent usage				0.004
No	58 (69.9)	42 (63.6)	16 (94.1)	
Yes	24 (28.9)	24 (36.4)	0 (0.0)	
Diagnosis				0.089
CS	36 (43.4)	32 (48.5)	4 (23.5)	
CS and FS and HNP	1 (1.2)	1 (1.5)	0 (0.0)	
CS and HNP	5 (6.0)	5 (7.6)	0 (0.0)	
CS and isthmic SPL	1 (1.2)	1 (1.5)	0 (0.0)	
FS	4 (4.8)	4 (6.1)	0 (0.0)	
FS and isthmic SPL	2 (2.4)	2 (3.0)	0 (0.0)	
HNP	33 (39.8)	20 (30.3)	13 (76.5)	
Facet cyst	1 (1.2)	1 (1.5)	0 (0.0)	
Operative type				0.022
Decompression (unilateral decompression)	7 (8.4)	6 (9.1)	1 (5.9)	
Decompression (bilateral, ULBD)	36 (43.4)	32 (48.5)	4 (23.5)	
Decompression (bilateral, ULBD) and discectomy	8 (9.6)	8 (12.1)	0 (0.0)	
Discectomy	32 (38.6)	20 (30.3)	12 (70.6)	
Index level				0.650
L1–2	1 (1.2)	1 (1.5)	0 (0.0)	
L2–3	7 (8.4)	7 (10.6)	0 (0.0)	
L3–4	19 (22.9)	16 (24.2)	3 (17.6)	
L4–5	40 (48.2)	29 (43.9)	11 (64.7)	
L5–S1	14 (16.9)	11 (16.7)	3 (17.6)	
T11–12	2 (2.4)	2 (3.0)	0 (0.0)	
Primary vs. Revision				0.458
Primary	63 (75.9)	47 (71.2)	16 (94.1)	
Revision	2 (2.4)	1 (1.5)	1 (5.9)	
PBV (L)	4.20 ± 0.79	4.17 ± 0.83	4.34 ± 0.59	0.425
Preop Hct (mL)	389.40 ± 45.66	380.21 ± 41.93	425.06 ± 42.92	<0.001
Postop Hct (mL)	366.41 ± 46.99	358.91 ± 44.20	395.53 ± 47.38	0.028
Hct average (mL)	377.90 ± 43.96	369.56 ± 40.43	410.29 ± 43.13	<0.001
TBL (mL)	257.72 ± 329.66	247.16 ± 346.88	298.71 ± 256.65	0.568
VBL (mL)	103.77 ± 59.96	97.71 ± 57.52	127.29 ± 65.16	<0.001
Operative time (min)	114.67 ± 32.75	124.26 ± 28.97	77.47 ± 15.41	<0.001
Intraoperative blood loss	36.22 ± 25.53	29.11 ± 23.11	63.82 ± 12.31	<0.001
Postop 1 day drain (mL)	37.41 ± 38.95	37.74 ± 27.18	36.12 ± 69.07	0.021
Postop 2 day drain (mL)	28.97 ± 25.76	29.40 ± 28.11	27.35 ± 14.47	0.491
Postop 3 day drain (mL)	13.21 ± 10.36	13.21 ± 10.36	0.00 ± 0.00	-
Total drain amount (mL)	67.55 ± 53.27	68.61 ± 50.14	63.47 ± 65.64	0.513

All continuous variables presented in mean ± SD. BESS, biportal endoscopic spine surgery; CS, central stenosis; FS, foraminal stenosis; HNP, herniation of nucleus pulposus; SPL, spondylolisthesis; ULBD, unilateral laminotomy and bilateral decompression; PBV, predicted blood volume, TBL, total blood loss; VBL, visible blood loss.

**Table 2:** Comparison of HBL between the BESS group and the open surgery group.

	HBL (mL)	t	*p*-Value
BESS group	149.44 ± 344.08	−0.302	0.764
Open surgery group	171.42 ± 243.93		

## Data Availability

The raw data supporting the conclusions of this article will be made available by the authors on request.

## Abbreviations

The following abbreviations are used in this manuscript:

BESS	Biportal endoscopic spine surgery
VBL	Visible blood loss
HBL	Hidden blood loss
TBL	Total blood loss
Hct	Hematocrit
MISS	Minimally invasive spine surgery
HNP	Herniation of nucleus pulposus
PBV	Predicted blood volume
FESS	Full endoscopic spine surgery
UBE	Unilateral biportal endoscopy
